# Hypertension in Latin America and the Caribbean: an analysis of recent progress and remaining challenges

**DOI:** 10.1590/2175-8239-JBN-2024-0245en

**Published:** 2025-07-04

**Authors:** Andrea Pio de Abreu

**Affiliations:** 1 Universidade de São Paulo, Faculdade de Medicina, Serviço de Nefrologia do Hospital das Clínicas, São Paulo, SP, Brazil.

**Keywords:** Hypertension, Latin America, Caribbean, Cardiovascular Diseases, Blood Pressure Monitoring, Ambulatory, Obesity, Renal Insufficiency, Chronic

## Abstract

This narrative review aims to present the current information on cardiovascular diseases, focusing on hypertension (HT) and associated comorbidities in Latin American and Caribbean (LAC) countries, and compare successful global studies on the subject. LAC countries have unique characteristics, including high socioeconomic inequality and unequal access to health and urban infrastructure. At the same time, urbanization and economic growth have contributed to the proliferation of unhealthy lifestyles. HT is the primary risk factor for cardiovascular morbidity and mortality, affecting between 20 and 40% of the population in this region. It is of utmost importance to address the alarmingly low rates of awareness, treatment, and control of HT. This is further compounded by the rising prevalence of patients with metabolic disorders. Obesity and HT are two pivotal drivers of the cardio-renal disease *continuum* because patients with uncontrolled cardiovascular risk in mid-life are likely to be at increased risk of clinical cardiovascular and chronic kidney disease in old age. A series of recommended actions include developing population-wide prevention and control programs, implementing opportunistic screening, and using out-of-office blood pressure measurements. It is imperative that primary care and treatment adherence are reinforced. Moreover, accessibility and optimal distribution of efficacious, cost-effective antihypertensive medications are of paramount importance. Insights from high-income countries should be effectively conveyed to LAC countries, respecting the particularities of the regions involved.

## Introduction

The objective of this review is to present the current information on cardiovascular diseases, with a particular focus on hypertension (HT) and associated comorbidities in Latin American and Caribbean (LAC) countries. In addition, we will draw parallels with global studies on the subject.

Firstly, the most recent reviews on cardiovascular diseases focusing on HT and published over the past 10 years will be discussed, as will be the sociodemographic particularities of LAC, which could explain the particular situation of HT in these countries. Subsequently, HT is examined in detail, including associated comorbidities and a review of existing global studies on the subject conducted in LAC countries. Finally, the challenges and potential solutions related to HT control are presented.

This is a narrative review of the literature and does not provide an exhaustive, comprehensive review of the literature; however, it should be helpful for a rich and meaningful summary of the updated work on HT. For that, a search was conducted in Pubmed, Medline, and Scopus databases using the keywords pertinent to the topic. The most recent articles published within the last 10 years were selected for analysis. The search was based on the impact factor of the articles and journal metrics, highlighting the LAC countries. After, we used the most recent article on HT as the central article and chose directly related articles on the topic using the ResearchRabbit software (https://www.researchrabbit.ai/) to create a mind map based on the above central article, later identifying all articles about HT in LAC countries. The aforementioned methodology was then employed to identify the most significant HT-related articles in LAC.

## Published Reviews on Cardiovascular Diseases in the last 10 Years

A systematic review conducted in 2015^1^ focused on low and middle-income countries (LMIC). The study revealed an overall prevalence of HT of approximately 32.3% in the LAC region. The prevalence estimates were markedly higher in the elderly cohort than in younger adults, with no obvious sex-based disparity. Moreover, individuals with lower formal education, those with a higher body mass index (BMI), and urban residents were more likely to have HT than their counterparts. A subgroup analysis revealed that the LAC region exhibited the highest prevalence of HT, while the Middle East and North Africa region had the lowest rate (26.9%)^1^. Subsequently, in 2019, a cross-sectional study utilizing population-based data from 44 LMICs was published^2^. The dataset comprised 1,100,507 participants, of whom 17.5% exhibited evidence of HT. Among those with HT, 73.6% had never undergone a blood pressure (BP) measurement, and only 10.3% had successfully controlled their HT. The study revealed that LAC countries exhibited the most favorable predicted performance based on gross domestic product (GDP) per capita, while countries in sub-Saharan Africa had the least favorable outcomes. Brazil, Costa Rica, Ecuador, and Peru, in particular, exhibited significantly better performance across all care cascade steps relative to the predicted outcomes based on GDP per capita.

In 2021, another systematic review presented a comprehensive analysis of the burden of multimor­bidity in the LAC region^3^. The authors reported that four in ten participants (25%) have multimorbidity with notable variability. In particular, HT awareness reached approximately 64% in urban Latin American regions. Once more, the prevalence estimates for HT were lower in rural areas of LAC countries^3^.

The most recent review on the epidemiology of cardiovascular diseases (CVD) in LAC countries was published in 2024^4^. Although HT is the main risk factor for CVD-related deaths in LAC countries for both sexes, its age-standardized prevalence is stable or has decreased in the region between 1980 and 2016. Again, the prevalence was lower in rural settings, probably due to fewer stress-related diseases. The management of HT in vulnerable populations is a significant challenge in LAC countries, and it is essential to consider the specific characteristics of the region. The article addresses the experiences of adult populations who frequently encounter biological and societal adversity from an early age, accelerated urbanization, and an aging population, in addition to low socioeconomic conditions and inequity. The study proposed strategies for fighting the cardiometabolic disease epidemic in LAC countries. These strategies were guided by high-quality local data and focused on evidence-based, population-wide, and individual-based strategy well adapted to the local context^4^.

## Socio-Demographic Characteristics of LAC Countries

LAC countries have a population of 662 million, representing 8.2% of the global population^5^. The region is characterized by a rapid demographic transition, followed by a significant decline in mortality and fertility rates from the 1950s to the current low levels. The region is expected to reach its maximum population in 2056, reaching 752 million people^5^. This will result in significant alterations to the region’s age structure. By 2100, the proportion of the population aged 60 or above in the area is expected to exceed that of Asia, North America, Oceania, and Africa^6^. This will bring new challenges, adding to the existing issues of inequalities in socioeconomic level, well-being, and access to health and urban infrastructure, among others^4^.

**Figure 1:**
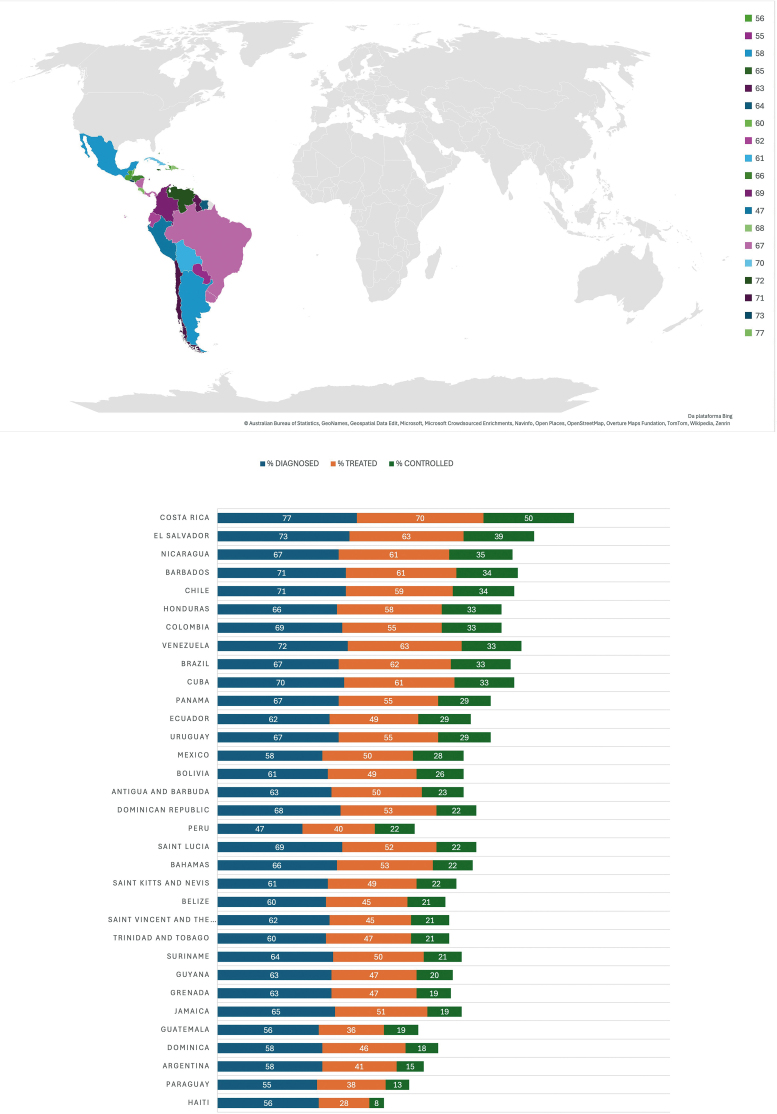
Representation of % diagnosed, treated, and controlled hypertension in Latin-American and Caribbean countries. The countries are in a decreasing order of % controlled HT. Age-standardized prevalence of HT among adults aged 30–79 years (2019). Data collected and modified from the WHO^9^.

Concurrent with this scenario, urbanization and economic growth have contributed to the proliferation of unhealthy lifestyles, including tobacco use, physical inactivity, and poor dietary habits. The Global Burden of Disease Study^7^ revealed that deaths across all LAC countries from non-communicable diseases (NCDs) including HT will increase. In 2019, Paraguay, Argentina, and Guatemala had the lowest rate of controlled HT in Latin America (between 13 and 19%; see Figure 1). Only 8% of the population in Haiti have controlled HT and only 28% were treated, followed by Dominica and Jamaica (controlled HT of 18% and 19%, respectively). Peru has the lowest rate of diagnosed HT in the population (47%), followed by Paraguay (55%). Finally, the highest rates of controlled HT were observed in El Salvador (39%) and Costa Rica (50%)^4^ (Figure 1). The high prevalence of HT and the low rates of awareness, treatment, and control in LAC countries explain why the number of premature cardiovascular deaths in this region is higher than in the USA and Europe^8^.

Latin American countries have a several of the ethnic, economic, geographic, and cultural charac­teristics that contribute to the elevated HT preva­lence. It is also acknowledged that socioeconomic inequalities associated with specific ethnicities constitute cardiovascular risk factors (CVRF). The unequal distribution of health services between urban and rural areas is a further factor contributing to the disparities in CVD prevalence and management. While the prevalence of HT has been declining in high-income countries, it has been increasing in low- and middle-income countries^10^. The extremely low rates of HT awareness, treatment, and control, particularly in patients with metabolic disorders, is a severe problem in LAC countries^10,11,12,13^. In addition to population growth and aging, the prevalence of five cardiometabolic risk factors (smoking, body mass index, diabetes, HT, and non-high-density lipoprotein cholesterol levels) can be used to predict the future burden of cardiovascular disease in LAC countries^8^.

Table 1 displays the percentage of controlled HT and Gini coefficient for LAC countries. The highest Gini coefficient is observed in Suriname, followed by Brazil, Colombia, and Panama, which are all around 50%. The Gini coefficient seems unrelated to controlled hypertension in these countries. Cuba has the lowest inequality level among LAC countries (0.38).

**Table 1:** Latin american and caribbean countries and % of controlled hypertension (HT). the countries are listed in decreasing order of % controlled hypertension. age-standardized prevalence of hypertension among adults aged 30–79 years (2019)

Countries	% Controlled HT	Gini coefficient*/year collected
Costa Rica	50	47.2 (2022)
El Salvador	39	38.8 (2022)
Nicaragua	35	46.2 (2014)
Barbados	34	NA
Chile	33	44.9 (2020)
Honduras	33	48.2 (2019)
Colombia	33	51.5 (2021)
Venezuela	33	39.0 (2011)
Brazil	33	52.9 (2021)
Cuba	33	0.38 (2000)
Panama	29	50.9 (2021)
Ecuador	29	45.5 (2022)
Uruguay	29	40.8 (2021)
Mexico	28	45.4 (2020)
Bolivia	26	40.9 (2021)
Antigua and Barbuda	23	NA
Dominican Republic	22	38.5 (2021)
Peru	22	40.2 (2021)
Saint Lucia	22	51.2 (2016)
Bahamas	22	NA
Saint Kitts and Nevis	22	NA
Belize	21	53.3 (1999)
Saint Vincent and the Grenadines	21	NA
Trinidad and Tobago	21	40.3 (1999)
Suriname	21	57.9 (1999)
Guyana	20	45.1 (1998)
Grenada	19	43.8 (2018)
Jamaica	19	35.0 (2016)
Guatemala	19	48.3 (2014)
Dominica	18	NA
Argentina	15	42.0 (2021)
Paraguay	13	45.1 (2022)
Haiti	8	41.1 (2012)

Abbreviations – HT, hypertension; NA, not available.

Notes – *The Gini coefficient is the most used measure of income distribution – the higher the Gini coefficient, the greater the income gap between the country’s richest and poorest people. The Gini coefficient helps identify high levels of income inequality, which can have several undesirable political and economic impacts. Source – World Population Review^14^ and Central Intelligence Agency^15^. Adapted from World Health Organization^9^.

## Epidemiology of Hypertension in LAC Countries

HT is responsible for more than two million deaths from cardiovascular disease annually in LAC countries, of which one million occur before 70 years of age^8^. Ischemic heart disease was the leading cause of death in 2019 in LAC countries, accounting for more deaths than any other NCD in both sexes. Stroke was the second leading cause of death in the same period. While crude mortality from stroke remained relatively stable between 1990 and 2019, with a slight decline of 3%, mortality from ischemic heart disease exhibited a notable increase of 19%. Risk factors that are more strongly associated with ischemic heart disease like diabetes are increasing rapidly in LAC countries^4^. Moreover, the region has the highest death rate from chronic kidney disease (CKD) worldwide, and CKD is the second leading cause of years of life lost^16^. BP, together with diabetes, is also a critical risk factor for CKD, which is a leading cause of death beyond NCD^17^.

In the Americas, over a quarter of adult women and four in ten adult men have HT, and the diagnosis, treatment, and control of the condition are suboptimal. In 2021, the World Health Organization (WHO) published an updated guideline for the pharmacological treatment of HT in adults^7^. This policy paper elucidates the instrumental role of the WHO Global HEARTS and the HEARTS in the Americas initiative in accelerating the implementation of the guideline (6,7). It offers detailed policy counsel for implementation and underscores the necessity for an overarching strategic approach to HT control. The authors recommend that health advocates and policymakers prioritize the prevention and control of HT to enhance the health and well-being of their populations and reduce cardiovascular disease disparities within and between populations of the Americas^6^.

Diagnosing and using off-patent medicines to treat HT at the primary-care level seems to be the most effective approach in reducing BP-related NCD deaths^17^, as has been implemented in high-income countries with effective HT programs. The Resolve to Save Lives initiative^18^ aims to reduce salt intake and treat HT using a simplified protocol at the primary care level in several low-income and middle-income countries. As the initiative is in its initial phases, it has not yet been evaluated, but if successful, this project could guide the design of future national programs.

In high-income countries, studies have generally reported an inverse association between HT and socioeconomic status (SES) at the individual level and area level^19^. In contrast, evidence of this association in Latin America remains comparatively scarce and conflicting. One study reported no association of HT with neighborhood- and city-level education in Argentina^20^. In Brazil, the prevalence of HT was significantly higher among residents living in census tracts with lower levels of education^21^, and the Federation Units in Brazil with a higher human development index had a higher prevalence of HT.

### HT and Associated Comorbidities

It is crucial to underscore that HT represents an independent risk factor for organ damage. The damage to target organs such as the brain, kidneys, heart, and vessels is associated with other metabolic risk factors that generate socio-economic impacts worldwide. Therefore, it is of utmost importance to note that these risk factors are both modifiable and preventable^22^. By 2035, based on current projections, it is estimated that a significant proportion of the urban population of LAC countries, ranging from 80 to 90 percent, will be affected by HT and obesity. The co-occurrence of these two significant cardiovascular risk factors may, in part, be attributed to the rapid socio-economic transitions occurring in this region, which promote the adoption of more sedentary lifestyles and less healthy dietary habits^12,23^. Furthermore, diabetes is the primary underlying cause of CKD in 36% of patients undergoing kidney replacement therapy (KRT), and HT, advanced age, obesity, and overweight are significant traditional risk factors.

Subclinical organ damage is an early-stage injury to organs that occurs without apparent symptoms in the heart, kidneys, brain, and blood vessels^8^. Regular screening for HT-mediated organ damage (HMOD) is essential. HT induces structural changes in the heart, including left ventricular hypertrophy (LVH), renal damage, and endothelial dysfunction. HT causes vascular changes due to endothelial dysfunction and arteriolar remodeling, initially manifesting as increased intima-media thickness (IMT) or small atheromatous plaques^24^. Prolonged exposure to HT leads to arterial stiffening, manifested by changes in the arterial media layer, such as elastin fragmentation and calcification, and finally, significantly increases the risk of stroke and cognitive decline^25^. Long-term high BP is a major risk factor for stroke, coronary artery disease, heart failure, atrial fibrillation, peripheral arterial disease, vision loss, chronic kidney disease, dementia, and even death^26^. High BP has long been recognized as a major health burden and particularly as a significant risk factor for stroke, cardiovascular disease, end-stage renal disease, and overall mortality that affects all segments of the population^27^.

In the LAC region, CKD is one of the most rapidly increasing NCD. Despite the widespread recognition of HT as a significant contributor to CKD, there is a paucity of comprehensive, quantitative research examining the burden of CKD attributable to HT. Specifically, in East Asia, Eastern Europe, tropical Latin America, and Western Sub-Saharan Africa, HT constituted the largest proportion of the disease burden of CKD. A global study demonstrated that the inequality-adjusted human development index was negatively correlated with the disease burden of CKD due to HT. This indicates that countries with less inequality in the three key dimensions of human development (health, education, and income) were more effective at reducing the disease burden of CKD due to HT. The findings indicate that in some developing countries, particularly those with limited resources, inadequate infrastructure, high costs of CKD treatment, limited labor force, and an absence of efficacious health policies, a comprehensive strategy is necessary to provide viable solutions for effectively monitoring kidney health and reducing the disease burden of CKD caused by HT^28^. Furthermore, up to 60% of hypertensive patients may have an abnormal lipid profile, whereas most patients with type 2 diabetes (DM-2) of a few years’ duration have high BP. The coexistence of HT and dyslipidemia increases by four times the risk of CVD and could increase more than 20-fold when multiple cardiometabolic risk factors (DM-2, obesity, and smoking) are present^23,29,30,31^.

The impact of BMI on HT is direct and positive. As for indirect effects, there are associations between race/skin color and HT mediated by socioeconomic position^32^. BMI represents the level of abdominal adiposity in different populations and is used to predict HT status^33^. Data representative of the populations of all world regions indicate that BMI and waist-to-height ratio (WHtR) are correlated at the individual level. However, at the same BMI level, WHtR was highest in South Asia, followed by LAC countries, and lowest in central and eastern Europe.

Finally, the development of kidney damage results from elevated BP. Hypertensive nephrosclerosis is the second leading cause of admission to chronic dialysis after diabetes mellitus^34^. The progression of renal disease appears to be associated with the degree of BP control. In Latin America, a registry including 20 countries representing 99% of the region’s population was utilized to monitor KRT for end-stage renal disease. KRT has increased in Latin America from 119 patients per million in 1991 to 660 patients per million in 2010^28,35,36^. Consequently, the prevention of comorbidities directly associated with HT would result in the prevention of a multitude of other diseases.

### Successful Programs Involving LAC Countries

To address this significant public health epidemic, 18 countries in the LAC region collaborated on the May Measurement Month (MMM) initiative in 2017 to raise awareness of high BP. The mean systolic BP (SBP) was 122.7 mmHg and mean diastolic BP (DBP) was 75.6 mmHg^37^. Following the application of the imputation methodology, it was determined that 42,328 participants (40.4%) had HT. The considerable number of participants identified with HT, coupled with the relatively large proportion of individuals on antihypertensive medication with uncontrolled HT, underscores the continued relevance of this annual event in the region. It highlights the necessity of raising awareness about preventing cardiovascular events^37^.

HEARTS program in the Americas is a regional program involving 32 countries in the LAC region with the Pan American Health Organization (PAHO) technical cooperation. It includes approximately 3000 health centers and covers approximately 30 million adults^6,19^. The goal is to improve HT control and promote secondary prevention of CVDs, emphasizing primary healthcare settings^9^. BP measurement is an essential component of continuous quality improvement of the initiative, which includes clinical training, the use of a protocol, and clinically validated devices. When the initiative was first launched, a small study showed that not only countries did not have the regulations necessary to ensure that only validated BP monitoring devices (BPMDs) were allowed in the market, but ministries of health and other health authorities had no record of the types of devices, brands, and models that were being used in primary health care facilities. As in other parts of the world, the market in Latin America is dominated by non-validated BPMDs^20^.

To date, the HEARTS in the Americas program has resulted in improvements to antihypertensive medication formularies and the establishment of pharmacological treatment protocols tailored to the specific needs of individual participating countries. This has resulted in a notable increase in HT control rates following the program’s implementation in these jurisdictions. The HEARTS in the Americas program may serve as a model not only for the Americas region, but also globally, with the potential to decrease the global burden of CVD^19^.

A recent study using unique data compiled and harmonized by the SALURBAL (Salud Urbana en America Latina/Urban Health in Latin America) project investigated how individual-level and area-level socio-economic status (SES) is associated with HT in adults from 230 cities in eight Latin American countries^19^. Gender differences were found in the relationship between individual education and HT, with higher individual-level education associated with lower odds of HT among women and higher odds among men. Second, higher sub-city-level education was positively associated with HT in both women and men. Third, higher city-level education was associated with lower odds of HT in both sexes in Peru. It is suggested that strategies to deal with the burden of HT in low-income countries should adopt equity-based and context-sensitive efforts^19^.

The ARTEMIS (International Ambulatory Blood Pressure Registry) is a telehealth initiative that monitors HT and cardiovascular risk globally^38^. This is the inaugural international registry of ambulatory BP monitoring (ABPM) that evaluates the actual degree of BP and cardiovascular risk control of hypertensive patients under the care of medical professionals in numerous countries (www.artemisnet.org). The project’s objectives include the establishment of a global network of centers engaged in ABPM data collection and the aggregation of a vast repository of clinical data from patients across diverse geographical regions. These patients must have basic clinical information available, in addition to at least one ABPM recording that meets predefined criteria^39^. The Artemis registry analyzed data from 14,143 patients from 27 countries from the 5 continents, including data from 1273 individuals from the American continent (Argentina, Brazil, Canada, Mexico, and Venezuela)^39^.

The CARMELA study, a cross-sectional survey designed to assess cardiovascular risk factors in adult populations aged 25–64 years from seven cities in South and North America, is another initiative in LAC countries^40^. Considerable variation in HT prevalence, awareness, treatment, and control was observed. For example, HT prevalence ranged from 9% (Quito) to 29% (Buenos Aires), undiagnosed HT ranged from 24% (Mexico City) to 47% (Lima), and treated and controlled HT ranged from 12% (Lima) to 41% (Mexico City)^40^. However, age-specific data showed that HT prevalences were similar in men but slightly higher in women. Since the CARMELA study was performed 14 years ago, HT prevalence and control have improved very little in Latin America. More recently, the Prospective Urban Rural Epidemiological (PURE) study reported that the prevalence of HT was 50.9% in Argentina, 52.6% in Brazil, and 46.6% in Chile among participants aged 35–70 years^11^. In addition, the study reported that awareness was lower in these countries compared to high-income countries.

Another study, the ELSA-Brasil cohort study, reported that the four-year incidence of HT was higher in individuals self-reported as Black race than in those self-reported as White race (21% versus 13%, respectively), and the prevalence of uncontrolled HT was also higher in the Black cohort (54.2% versus 39.0%, respectively)^32^. A recent ELSA study in Brazil reported that physical activity was directly correlated to a lower risk of developing HT^41^.

### Challenges

The most frequently described problems related to HT are the health system’s barriers that hinder comprehensive and equitable access to medical care and medications and the absence of educational programs and personalized interventions that improve adherence to treatment and lifestyle changes. The economic factor is critical in Latin America and impedes access to the health system and lifestyle changes due to cost related to transportation, medical appointments, and medications. The barriers detected affect all dimensions of health care^42^.

Latin American HT guidelines^8^, tailored to the needs of countries of Central and South America, should be applied and adopted by most Latin American physicians^21^. The knowledge and implementation of the guidelines are one of the most significant challenges of HT societies in Latin America, such as the Central American and Caribbean Society of Arterial HT (SCCH), the Latin American Society of HT (LASH), and the Inter-American Society of Cardiology (SIAC). The SIAC document reinforces all proposals by the LASH guidelines concerning the therapeutic approach and pharmacological recommendations for patients with HT to achieve better HT control in the Central American and Caribbean area, and thus improve prognosis of cardiovascular disease in the region^6,8,13,22^.

In order to achieve the primary objectives of antihypertensive treatment, namely a reduction in cardiovascular and renal morbidity and mortality, as well as s better quality of life, it is imperative that treatment be promptly initiated (ideally before significant organ damage), efficacious (attaining the prescribed targets), and maintained over time (with targets being consistently attained). Achieving these goals is a significant challenge on a global scale, with Latin America facing particularly formidable obstacles.

The challenges for LAC countries, one of the world regions with the most significant disparities in socioeconomic conditions, are related to contrasts in healthcare access. The proportion of people living in poverty varies considerably between different areas of LAC countries^6^. Furthermore, significant disparities exist regarding the structure, accessibility, quality, and funding of national health systems. Some health systems are entirely public, such as the Cuban system, or predominantly public, such as those seen in Jamaica^10^. Additionally, there are many mixed systems, such as those in Brazil, Ecuador, and Peru. In the region, there are significant disparities in the equity of medical services. In countries such as Bolivia, Peru, and Guatemala, only 19.8%, 14.3%, and 9.3%, respectively, of individuals with low income have access to medical services^34^.

It is of the utmost importance to accurately measure BP in an office setting to diagnose and manage HT effectively. This is more pressing in Latin America than anywhere else in the world^38^. In Latin America, office BP (OBP) measurement is predominantly used for screening, diagnosing, monitoring, and following up on HT. Nevertheless, awareness of OBP limitations has grown, and evidence of the value of ambulatory BP monitoring (ABPM) as a complement to OBP in the clinical approach to the hypertensive patient has accumulated and represents the gold standard for BP measurements^38,39,43^. The daytime and nighttime BP patterns offered by 24-h ABPM in the diagnostic, prognostic, and therapeutic management of HT has been gaining attention worldwide. In Latin American countries, most scientific societies of HT and/or cardiology have published guidelines for HT care, three of which include a dedicated section on ABPM. However, in Latin America, the access to ABPM is frequently constrained to urban areas with a substantial population. Additionally, the costs associated with this technology can impede its utilization by patients with limited financial resources, especially in LAC countries^38,43,44,45,46^. Another critical issue in LAC countries is that each country has its own national regulatory agency for the validation of medical devices. A uniform policy across LAC countries for using the same international validation protocol should contribute to reducing the use of inaccurate BP monitors in the market^8^.

### Perspectives

Following the World Heart Federation Roadmap for HT^47^, a series of actions have been proposed, including the development of population-wide prevention and control programs, the implementation of opportunistic screening, and out-of-office BP measurements. The strengthening of primary care and a greater focus on task sharing and team-based care are essential. The delivery of people-centered care and patient and care education, together with the promotion of treatment adherence, are critical^47^. All of the above depend upon the availability and effective distribution of good quality, evidence-based, inexpensive BP-lowering agents.

The use of telemedicine, together with BP measurements outside the medical office, could improve access to the health system, adherence, and maintenance of hypertensive treatment^48^. Introducing BP telemonitoring systems could enhance routine screening and management of HT after medical office diagnosis of HT and consequently reduce the adverse impact on the global disease burden in Latin America. A transcendent issue is the need to make the population aware of the benefits of taking BP to avoid HT complications and thus promote the creation of teleconsultation mechanisms for the follow-up of patients already diagnosed with HT.

Artificial intelligence systems are also currently used to develop novel BP monitoring technologies consisting of wearable sensors, either cuff-based or cuffless devices that use mechanical and optical sensors to determine features of the blood pulse waveform shape, such as tonometry, photoplethysmography, and capacitance, to estimate BP^8^.

Finally, lessons learned from successful experiences in high-income countries can be adapted regionally and foster the exchange of resources, technology, and knowledge between low- and middle-income countries. The main goal is achieving the goals of the United Nations 2030 Agenda for Sustainable Development^4^.

## Data Availability

No new data were generated or analyzed in this study.
