# Supplementary material for: Hypertension in Latin America and the Caribbean: an analysis of recent progress and remaining challenges
Source: J Bras Nefrol. 2025 Jul 4;47(3):e20240245. doi: 10.1590/2175-8239-JBN-2024-0245en (PMC12227124; doi:10.1590/2175-8239-JBN-2024-0245en)
Supplement: Translation

# Translation: Hipertensão na América Latina e no Caribe: uma análise do recente progresso e dos desafios remanescentes

**DOI:** 10.1590/2175-8239-JBN-2024-0245pt

**Authors:** Andrea Pio de Abreu

**Affiliations:** 1 Universidade de São Paulo, Faculdade de Medicina, Serviço de Nefrologia do Hospital das Clínicas, São Paulo, SP, Brasil.

**Keywords:** Hipertensão, América Latina, Região do Caribe, Doenças Cardiovasculares, Monitorização Ambulatorial da Pressão Arterial, Obesidade, Insuficiência Renal Crônica

## Abstract

Esta revisão narrativa tem como objetivo apresentar informações atuais sobre doenças cardiovasculares, com foco na hipertensão arterial (HA) e comorbidades associadas nos países da América Latina e do Caribe (ALC), além de comparar estudos globais bem-sucedidos sobre o tema. Os países da ALC apresentam características únicas, incluindo elevada desigualdade socioeconômica e acesso desigual à saúde e à infraestrutura urbana. Ao mesmo tempo, a urbanização e o crescimento econômico contribuíram para a proliferação de estilos de vida pouco saudáveis. A hipertensão é o principal fator de risco para a morbimortalidade cardiovascular, afetando entre 20–40% da população dessa região. É de extrema importância abordar as taxas alarmantemente baixas de conscientização, tratamento e controle da HA. Isso é ainda mais agravado pela crescente prevalência de pacientes com distúrbios metabólicos. A obesidade e a HA são dois fatores essenciais do *continuum* da doença cardiorrenal, uma vez que pacientes com risco cardiovascular não controlado na meia-idade tendem a apresentar maior risco de doença cardiovascular clínica e doença renal crônica durante o envelhecimento. Uma série de ações recomendadas inclui o desenvolvimento de programas de prevenção e controle em nível populacional, a implementação de triagem oportunista e o uso de medições de pressão arterial fora do consultório. É imperativo que a atenção primária e a adesão ao tratamento sejam reforçadas. Além disso, acessibilidade e distribuição ideal de medicamentos anti-hipertensivos eficazes e custo-efetivos são de suma importância. As percepções dos países de alta renda devem ser transmitidas de forma eficaz aos países da ALC, respeitando as particularidades das regiões envolvidas.

## Introdução

O objetivo desta revisão é apresentar as informações atuais sobre doenças cardiovasculares, com foco especial na hipertensão (HA) e comorbidades associadas nos países da América Latina e do Caribe (ALC). Além disso, serão traçados paralelos com estudos globais sobre o assunto.

Primeiramente, serão discutidas as revisões mais recentes sobre doenças cardiovasculares com foco na HA, publicadas nos últimos 10 anos, bem como as particularidades sociodemográficas da ALC, que podem explicar a situação específica da HA nesses países. Posteriormente, a HA é examinada em detalhes, incluindo as comorbidades associadas e uma revisão dos estudos globais existentes sobre o tema, realizados em países da ALC. Por fim, são apresentados os desafios e as possíveis soluções relacionadas ao controle da HA.

Esta é uma revisão narrativa da literatura, que não oferece uma análise exaustiva; no entanto, deve ser útil para um resumo abrangente e significativo do trabalho atualizado sobre a HA. Para isso, foi realizada uma busca nos bancos de dados Pubmed, Medline e Scopus, utilizando as palavras-chave pertinentes ao tópico. Foram selecionados para análise os artigos mais recentes publicados nos últimos 10 anos. A pesquisa foi baseada no fator de impacto dos artigos e nas métricas dos periódicos, com destaque para os países da ALC. Em seguida, utilizamos o artigo mais recente sobre HA como artigo central e selecionamos artigos diretamente relacionados ao tema por meio do software ResearchRabbit (https://www.researchrabbit.ai/), com o objetivo de criar um mapa mental baseado nesse artigo central, identificando em seguida todos os artigos sobre HA nos países da América Latina e do Caribe. A metodologia supracitada foi então empregada para identificar os artigos mais significativos relacionados à HA na ALC.

## Revisões Publicadas Sobre Doenças Cardiovasculares nos Últimos 10 Anos

Uma revisão sistemática realizada em 2015^1^ concentrou-se em países de baixa e média renda (PBMR). O estudo revelou uma prevalência geral de HA de aproximadamente 32,3% na região da ALC. As estimativas de prevalência foram acentuadamente mais elevadas na coorte de idosos em comparação aos adultos mais jovens, sem disparidade evidente baseada no sexo. Além disso, indivíduos com menor nível de educação formal, aqueles com maior índice de massa corporal (IMC) e os residentes de áreas urbanas apresentaram maior probabilidade de ter HA do que seus pares. Uma análise de subgrupo revelou que a região da ALC apresentou a maior prevalência de HA, enquanto a região do Oriente Médio e Norte da África registrou a menor taxa (26,9%)^1^. Posteriormente, em 2019, foi publicado um estudo transversal utilizando dados de base populacional de 44 PBMR^2^. O conjunto de dados incluiu 1.100.507 participantes, dos quais 17,5% apresentaram evidências de HA. Entre aqueles com HA, 73,6% nunca haviam se submetido a uma aferição da pressão arterial (PA), e somente 10,3% tinham conseguido controlar com sucesso a condição. O estudo revelou que os países da ALC apresentaram o desempenho previsto mais favorável com base no produto interno bruto (PIB) per capita, enquanto os países da África Subsaariana registraram os desfechos menos favoráveis. Brasil, Costa Rica, Equador e Peru, em particular, apresentaram desempenho significativamente superior em todas as etapas da cascata de cuidado em relação aos desfechos previstos com base no PIB per capita.

Em 2021, outra revisão sistemática apresentou uma análise abrangente da carga de multimorbidade na região da ALC^3^. Os autores relataram que quatro em cada dez participantes (25%) têm multimorbidade com variabilidade notável. Em particular, a conscientização sobre a HA alcançou aproximadamente 64% nas regiões urbanas da América Latina. Mais uma vez, as estimativas de prevalência da HA foram menores nas áreas rurais dos países da ALC^3^.

A revisão mais recente sobre a epidemiologia das doenças cardiovasculares (DCV) nos países da ALC foi publicada em 2024^4^. Embora a HA seja o principal fator de risco para óbitos relacionados a DCV na região para ambos os sexos, sua prevalência padronizada por idade manteve-se estável ou sofreu redução entre 1980 e 2016. Novamente, a prevalência foi menor em ambientes rurais, provavelmente devido à menor incidência de doenças relacionadas ao estresse. O manejo da HA em populações vulneráveis constitui um desafio significativo nos países da ALC, sendo essencial considerar as características específicas da região. O artigo aborda as experiências de populações adultas que frequentemente enfrentam adversidades biológicas e sociais desde a infância, além da urbanização acelerada e do envelhecimento da população, somados a condições socioeconômicas precárias e à desigualdade. O estudo propôs estratégias para combater a epidemia de doenças cardiometabólicas nos países da ALC. Essas estratégias foram orientadas por dados locais de alta qualidade e se concentraram em estratégias baseadas em evidências, com abrangência populacional e individual, devidamente adaptadas ao contexto local^4^.

## Características Sociodemográficas dos Países da ALC

Os países da ALC possuem uma população de 662 milhões de habitantes, o que representa 8,2% da população mundial^5^. A região é caracterizada por uma rápida transição demográfica, seguida por um declínio significativo nas taxas de mortalidade e fertilidade desde a década de 1950 até os baixos níveis atuais. Espera-se que a região atinja seu pico populacional em 2056, chegando a 752 milhões de pessoas^5^. Isso resultará em alterações significativas na estrutura etária da região. Até 2100, espera-se que a proporção da população com 60 anos ou mais na área exceda a da Ásia, América do Norte, Oceania e África^6^. Isso trará novos desafios, somando-se às questões existentes de desigualdades no nível socioeconômico, no bem-estar e no acesso à saúde e à infraestrutura urbana, entre outros^4^.

Paralelamente a esse cenário, a urbanização e o crescimento econômico contribuíram para a proliferação de estilos de vida pouco saudáveis, incluindo o uso de tabaco, a inatividade física e os maus hábitos alimentares. O estudo *Global Burden of Disease* ^7^ revelou que os óbitos por doenças crônicas não transmissíveis (DNCTs), incluindo a HA, aumentarão em todos os países da ALC. Em 2019, Paraguai, Argentina e Guatemala apresentaram a menor taxa de HA controlada na América Latina (entre 13 e 19%; ver Figura 1). Somente 8% da população do Haiti têm HA controlada e apenas 28% receberam tratamento, seguido por Dominica e Jamaica HA controlada de 18% e 19%, respectivamente). O Peru tem a menor taxa de diagnóstico de HA na população (47%), seguido pelo Paraguai (55%). Por fim, as taxas mais elevadas de HA controlada foram observadas em El Salvador (39%) e Costa Rica (50%)^4^ (Figura 1). A alta prevalência de HA e as baixas taxas de conscientização, tratamento e controle nos países da ALC explicam porque o número de mortes cardiovasculares prematuras nessa região é maior do que nos EUA e na Europa^8^.

**Figura 1:** Representação da % de hipertensão diagnosticada, tratada e controlada nos países da América Latina e do Caribe. Os países estão em ordem decrescente de % de HT HA controlada. Prevalência padronizada de HA por idade, entre adultos de 30 a 79 anos (2019). Dados coletados e modificados da OMS^9^.

Os países da América Latina apresentam várias características étnicas, econômicas, geográficas e culturais que contribuem para a prevalência elevada de HA. Também é reconhecido que as desigualdades socioeconômicas associadas a etnias específicas constituem fatores de risco cardiovasculares (FRCV). A distribuição desigual dos serviços de saúde entre as áreas urbanas e rurais é outro fator que contribui para as disparidades na prevalência e no manejo da DCV. Embora a prevalência de HA tenha diminuído em países de alta renda, observa-se um aumento em países de baixa e média renda^10^. As taxas extremamente baixas de conscientização, tratamento e controle da HA, especialmente em pacientes com distúrbios metabólicos, é um problema grave nos países da ALC^10,11,12,13^. Além do crescimento e do envelhecimento da população, a prevalência de cinco fatores de risco cardiometabólicos (tabagismo, índice de massa corporal, diabetes, HA e níveis de colesterol não-HDL) pode ser utilizada para prever a carga futura de doenças cardiovasculares nos países da ALC^8^.

A Tabela 1 exibe a porcentagem de HA controlada e o coeficiente de Gini para os países da ALC. O maior coeficiente de Gini foi observado no Suriname, seguido por Brasil, Colômbia e Panamá, todos em torno de 50%. O coeficiente de Gini parece não estar relacionado à hipertensão controlada nesses países. Cuba apresenta o menor nível de desigualdade entre os países da ALC (0,38).

**Tabela 1:** Países da américa latina e do caribe e % de hipertensão (HA) controlada. Os países estão listados em ordem decrescente de % de hipertensão controlada. Prevalência padronizada por idade de hipertensão entre adultos de 30 a 79 anos (2019)

Países	% HA controlada	Coeficiente de gini*/ano coletado
Costa Rica	50	47,2 (2022)
El Salvador	39	38,8 (2022)
Nicarágua	35	46,2 (2014)
Barbados	34	NA
Chile	33	44,9 (2020)
Honduras	33	48,2 (2019)
Colômbia	33	51,5 (2021)
Venezuela	33	39,0 (2011)
Brasil	33	52,9 (2021)
Cuba	33	0,38 (2000)
Panamá	29	50,9 (2021)
Equador	29	45,5 (2022)
Uruguai	29	40,8 (2021)
México	28	45,4 (2020)
Bolívia	26	40,9 (2021)
Antígua e Barbuda	23	NA
República Dominicana	22	38,5 (2021)
Peru	22	40,2 (2021)
Santa Lúcia	22	51,2 (2016)
Bahamas	22	NA
São Cristóvão e Névis	22	NA
Belize	21	53,3 (1999)
São Vicente e Granadinas	21	NA
Trinidade e Tobago	21	40,3 (1999)
Suriname	21	57,9 (1999)
Guiana	20	45,1 (1998)
Granada	19	43,8 (2018)
Jamaica	19	35,0 (2016)
Guatemala	19	48,3 (2014)
Dominica	18	NA
Argentina	15	42,0 (2021)
Paraguai	13	45,1 (2022)
Haiti	8	41,1 (2012)

Abreviações – HA, hipertensão; NA, não disponível.

Notas – *O coeficiente de Gini é a medida mais utilizada de distribuição de renda – quanto maior o coeficiente de Gini, maior a diferença de renda entre os mais ricos e os mais pobres do país. O coeficiente de Gini ajuda na identificação de altos níveis de desigualdade de renda, que podem ter diversos impactos políticos e econômicos indesejáveis. Fonte – *World Population Review* ^14^ e Agência Central de Inteligência^15^. Adaptado da Organização Mundial da Saúde^9^.

## Epidemiologia da Hipertensão nos Países da ALC

A HA é responsável por mais de dois milhões de óbitos por doenças cardiovasculares anualmente nos países da ALC, dos quais um milhão ocorrem antes dos 70 anos de idade^8^. A cardiopatia isquêmica foi a principal causa de óbito em 2019 na região, sendo responsável por mais óbitos do que qualquer outra DCNT em ambos os sexos. O AVC foi a segunda principal causa de óbito no mesmo período. Embora a mortalidade bruta por AVC tenha permanecido relativamente estável entre 1990 e 2019, com um leve declínio de 3%, a mortalidade por cardiopatia isquêmica apresentou um aumento expressivo de 19%. Os fatores de risco que estão mais fortemente associados à cardiopatia isquêmica, como o diabetes, estão aumentando rapidamente nos países da ALC^4^. Além disso, a região apresenta a maior taxa de mortalidade por doença renal crônica (DRC) em todo o mundo, sendo a DRC a segunda principal causa de anos de vida perdidos^16^. A HA, juntamente com o diabetes, também é um fator de risco crítico para a DRC, que representa a principal causa de óbito além das DCNTs^17^.

Nas Américas, mais de um quarto das mulheres adultas e quatro em cada dez homens adultos têm HA, e o diagnóstico, o tratamento e o controle da condição estão abaixo do ideal. Em 2021, a Organização Mundial da Saúde (OMS) publicou uma diretriz atualizada para o tratamento farmacológico da HA em adultos^7^. Este documento de política elucida o papel fundamental da iniciativa *Global HEARTS* da OMS e HEARTS nas Américas na aceleração da implementação da diretriz (6,7). Ele oferece recomendações políticas detalhadas para a implementação e ressalta a necessidade de uma abordagem estratégica abrangente para o controle da HA. Os autores recomendam que os defensores da saúde e os formuladores de políticas priorizem a prevenção e o controle da HA com o objetivo de promover a saúde e o bem-estar de suas populações e reduzir as disparidades de doenças cardiovasculares dentro e entre as populações das Américas^6^.

O diagnóstico e o uso de medicamentos não patenteados para o tratamento da HA no nível da atenção primária parecem ser a abordagem mais eficaz para reduzir as mortes por DCNT relacionadas à PA^17^, conforme tem sido implementado em países de alta renda com programas eficazes de controle de HA. A iniciativa *Resolve to Save Lives* ^18^ tem como objetivo reduzir a ingestão de sal e tratar a HA por meio de um protocolo simplificado no nível de atenção primária em diversos países de baixa e média renda. Como a iniciativa está em suas fases iniciais, ela ainda não foi avaliada, mas, se bem-sucedida, esse projeto poderá orientar a elaboração de futuros programas nacionais.

Em países de alta renda, os estudos geralmente relatam uma associação inversa entre HA e nível socioeconômico (NSE) em nível individual e regional^19^. Em contrapartida, as evidências dessa associação na América Latina permanecem comparativamente escassas e conflitantes. Um estudo não relatou nenhuma associação entre HA e educação nos âmbitos municipal e de bairro na Argentina^20^. No Brasil, a prevalência de HA foi significativamente mais elevada entre os residentes de setores censitários com níveis mais baixos de escolaridade^21^, e as Unidades Federativas do Brasil com maior Índice de Desenvolvimento Humano apresentaram maior prevalência de HA.

### HA e Comorbidades Associadas

É crucial ressaltar que a HA representa um fator de risco independente para lesão de órgãos. As lesões de órgãos-alvo, como cérebro, rins, coração e vasos, estão associadas a outros fatores de risco metabólicos que geram impactos socioeconômicos em uma escala mundial. Portanto, é de extrema importância observar que esses fatores de risco são modificáveis e evitáveis^22^. Com base nas projeções atuais, estima-se que, até 2035, uma proporção significativa da população urbana dos países da ALC, variando de 80 a 90%, será afetada pela HA e obesidade. A ocorrência desses dois importantes fatores de risco cardiovascular pode, em parte, ser atribuída às rápidas transições socioeconômicas que ocorrem nessa região, as quais promovem a adoção de estilos de vida mais sedentários e hábitos alimentares menos saudáveis^12,23^. Além disso, o diabetes é a principal causa subjacente da DRC em 36% dos pacientes submetidos à terapia renal substitutiva (TRS), sendo a HT, a idade avançada, a obesidade e o sobrepeso fatores de risco tradicionais significativos.

A lesão subclínica de órgãos é uma lesão em estágio inicial que ocorre sem sintomas aparentes no coração, nos rins, no cérebro e nos vasos sanguíneos^8^. A triagem regular para lesão de órgãos mediada pela HA (HMOD, do inglês *HT-mediated organ damage*) é essencial. A HA induz alterações estruturais no coração, incluindo hipertrofia ventricular esquerda (HVE), dano renal e disfunção endotelial. A HA também causa alterações vasculares devido à disfunção endotelial e ao remodelamento arteriolar, manifestando-se inicialmente como aumento da espessura da camada íntima-média (IMT, do inglês *intima-media thickness*) ou como pequenas placas de ateroma^24^. A exposição prolongada à HA leva ao enrijecimento arterial, manifestado por alterações na camada média das artérias, tais como fragmentação da elastina e calcificação e, por fim, aumenta significativamente o risco de AVC e declínio cognitivo^25^. HA por longo prazo constitui um fator de risco importante para AVC, doença arterial coronariana, insuficiência cardíaca, fibrilação atrial, doença arterial periférica, perda de visão, DRC, demência e até mesmo óbito^26^. A HA é, há muito, reconhecida como um importante problema de saúde e, particularmente, como um fator de risco significativo para acidente vascular cerebral, doença cardiovascular, doença renal em estágio terminal e mortalidade geral, afetando todos os segmentos da população^27^.

Na região da ALC, a DRC é uma das DCNT com crescimento mais rápido. Apesar do amplo reconhecimento da HA como um fator contribuinte significativo para a DRC, há uma escassez de pesquisas abrangentes e quantitativas que examinem a carga da DRC atribuível à HA. Especificamente, no Leste Asiático, Europa Oriental, América Latina tropical e África Subsaariana Ocidental, a HA constituiu a maior proporção da carga de doença na DRC. Um estudo global demonstrou que o Índice de Desenvolvimento Humano ajustado à desigualdade foi negativamente correlacionado com a carga de doença da DRC decorrente da HA. Isso indica que os países com menor desigualdade nas três principais dimensões do desenvolvimento humano (saúde, educação e renda) foram mais eficazes na redução da carga de doença da DRC devido à HA. Os achados indicam que, em alguns países em desenvolvimento, especialmente aqueles com recursos limitados, infraestrutura inadequada, altos custos relacionados ao tratamento da DRC, força de trabalho limitada e ausência de políticas de saúde eficazes, é necessária uma estratégia abrangente para fornecer soluções viáveis para o monitoramento eficaz da saúde renal e para a redução da carga da DRC causada pela HA^28^. Além disso, até 60% dos pacientes com HA podem apresentar um perfil lipídico anormal, enquanto a maioria dos pacientes com diabetes tipo 2 (DM2) com poucos anos de duração apresenta PA elevada. A coexistência de HA e dislipidemia aumenta em quatro vezes o risco de DCV e pode aumentar em mais de 20 vezes quando múltiplos fatores de risco cardiometabólicos (DM2, obesidade e tabagismo) estão presentes^23,29–31^.

O impacto do IMC na HA é direto e positivo. Quanto aos efeitos indiretos, há associações entre raça/cor da pele e HA mediadas pela posição socioeconômica^32^. O IMC representa o nível de adiposidade abdominal em diferentes populações e é utilizado como preditor do status da HA^33^. Os dados representativos das populações de todas as regiões do mundo indicam que o IMC e a razão cintura/estatura (RCE) estão correlacionados em nível individual. No entanto, no mesmo nível de IMC, a RCE foi mais elevada no sul da Ásia, seguida pelos países da ALC, e mais baixa na Europa Central e Oriental.

Por fim, o desenvolvimento de lesão renal resulta da PA elevada. A nefroesclerose hipertensiva é a segunda causa principal de admissão à diálise crônica, depois do diabetes mellitus^34^. A progressão da doença renal parece estar associada ao grau de controle da PA. Na América Latina, um registro que incluiu 20 países, representando 99% da população da região, foi utilizado para monitorar a TRS para doença renal em estágio terminal. A TRS aumentou na América Latina, passando de 119 pacientes por milhão em 1991 para 660 pacientes por milhão em 2010^28,35,36^. Consequentemente, a prevenção de comorbidades diretamente associadas à HA resultaria na prevenção de uma infinidade de outras doenças.

### Programas BEM-Sucedidos Envolvendo Países da ALC

Para abordar essa significativa epidemia de saúde pública, 18 países da região da ALC colaboraram com a iniciativa do Mês de Maio da Medição (MMM), em 2017, a fim de aumentar a conscientização sobre a PA elevada. A PA sistólica média (PAS) foi de 122,7 mmHg e a PA diastólica média (PAD) foi de 75,6 mmHg^37^. Após a aplicação da metodologia de imputação, determinou-se que 42.328 participantes (40,4%) tinham HA. O número considerável de participantes identificados com HA, juntamente com a proporção relativamente alta de indivíduos em uso de medicação anti-hipertensiva com HA não controlada, ressalta a relevância contínua desse evento anual na região. Ele destaca a necessidade de aumentar a conscientização sobre a prevenção de eventos cardiovasculares^37^.

O programa HEARTS nas Américas é um programa regional que envolve 32 países da região da ALC com a cooperação técnica da Organização Pan-Americana da Saúde (OPAS). Ele abrange cerca de 3.000 centros de saúde e cobre aproximadamente 30 milhões de adultos^6,19^. Seu objetivo é melhorar o controle da HA e promover a prevenção secundária das DCVs, com ênfase nos ambientes de atenção primária à saúde^9^. A medição da PA é um componente essencial da melhoria contínua da qualidade da iniciativa, que inclui treinamento clínico, o uso de um protocolo e dispositivos clinicamente validados. Quando a iniciativa foi inicialmente lançada, um estudo de pequena escala demonstrou que, além de os países não possuírem as regulamentações necessárias para garantir que apenas dispositivos de monitoramento da PA (DMPA) validados fossem permitidos no mercado, os ministérios da saúde e outras autoridades sanitárias não dispunham de nenhum registro dos tipos de dispositivos, marcas e modelos que estavam sendo utilizados nas unidades de atenção primária à saúde. Assim como em outras partes do mundo, o mercado na América Latina é dominado por DMPA não validados^20^.

Até o momento, o programa HEARTS nas Américas resultou em melhorias nos formulários de medicamentos anti-hipertensivos e no estabelecimento de protocolos de tratamento farmacológico adaptados às necessidades específicas de cada país participante. Isso resultou em um aumento notável nas taxas de controle da HA após a implementação do programa nessas jurisdições. O programa HEARTS nas Américas pode servir de modelo não apenas para a região das Américas, mas também globalmente, com o potencial de reduzir a carga global de DCV^19^.

Um estudo recente utilizando dados exclusivos compilados e harmonizados pelo projeto SALURBAL (Salud Urbana en America Latina/Saúde Urbana na América Latina) investigou como o nível socioeconômico (NSE), tanto em nível individual quanto regional, está associado à HA em adultos de 230 cidades em oito países da América Latina^19^. Diferenças de sexo foram encontradas na relação entre educação individual e HA, com maior escolaridade em nível individual associada a menores chances de HA entre as mulheres e maiores chances entre os homens. Em segundo lugar, níveis mais elevados de escolaridade em nível submunicipal foram positivamente associados à HA tanto em mulheres quanto em homens. Terceiro, a educação mais elevada em nível municipal esteve associada a menores chances de HA em ambos os sexos no Peru. Sugere-se que as estratégias para lidar com o ônus da HA em países de baixa renda devem adotar esforços baseados na equidade e sensíveis ao contexto^19^.

O ARTEMIS (Registro Internacional de Pressão Arterial Ambulatorial) é uma iniciativa de telessaúde que monitora a HA e o risco cardiovascular mun­dialmente^38^. Esse é o primeiro registro internacional de monitorização ambulatorial da PA (MAPA) que avalia o grau real de controle da PA e do risco cardiovascular de pacientes hipertensos sob os cuidados de profissionais médicos em diversos países (www.artemisnet.org). Os objetivos do projeto incluem o estabelecimento de uma rede global de centros envolvidos na coleta de dados de MAPA e a agregação de um amplo repositório de dados clínicos de pacientes de diversas regiões geográficas. Esses pacientes devem dispor de informações clínicas básicas, além de pelo menos um registro de MAPA que preencha critérios predefinidos^39^. O registro Artemis analisou dados de 14.143 pacientes de 27 países dos 5 continentes, incluindo dados de 1.273 indivíduos do continente americano (Argentina, Brasil, Canadá, México e Venezuela)^39^.

O estudo CARMELA, uma pesquisa transversal desenhada para avaliar fatores de risco cardiovascular em populações adultas com idades entre 25 e 64 anos, de sete cidades das Américas do Sul e do Norte, é outra iniciativa nos países da ALC^40^. Observou-se uma variação considerável na prevalência, conscientização, tratamento e controle da HA. Por exemplo, a prevalência de HA variou de 9% (Quito) a 29% (Buenos Aires), a HA não diagnosticada variou de 24% (Cidade do México) a 47% (Lima), e a HA tratada e controlada variou de 12% (Lima) a 41% (Cidade do México)^40^. No entanto, dados específicos por idade mostraram que as prevalências de HA foram semelhantes entre os homens, mas ligeiramente mais elevadas entre as mulheres. Desde a realização do estudo CARMELA, há 14 anos, a prevalência e o controle da HA melhoraram muito pouco na América Latina. Mais recentemente, o estudo *Prospective Urban Rural Epidemiological* (PURE) relatou que a prevalência de HA foi de 50,9% na Argentina, 52,6% no Brasil e 46,6% no Chile entre participantes de 35 a 70 anos^11^. Além disso, o estudo indicou que a conscientização foi menor nesses países em comparação aos países de alta renda.

Outro estudo, o estudo longitudinal ELSA-Brasil, relatou que a incidência de HA ao longo de quatro anos foi maior em indivíduos que se autodeclararam pretos em comparação com aqueles que se autodeclararam brancos (21% versus 13%, respectivamente), e a prevalência de HA não controlada também foi superior na coorte preta (54,2% versus 39,0%, respectivamente)^32^. Um estudo ELSA recente no Brasil relatou que a atividade física esteve diretamente correlacionada a um menor risco de desenvolvimento de HA^41^.

### Desafios

Os problemas mais frequentemente descritos rela­cionados à HA são as barreiras do sistema de saúde, que dificultam o acesso abrangente e equitativo à atenção médica e aos medicamentos, e a ausência de programas educacionais e intervenções personalizadas que promovam a adesão ao tratamento e mudanças no estilo de vida. O fator econômico é crítico na América Latina, impedindo o acesso ao sistema de saúde e às mudanças no estilo de vida devido aos custos relacionados a transporte, consultas médicas e medicamentos. As barreiras identificadas afetam todas as dimensões dos cuidados com a saúde^42^.

As diretrizes latino-americanas de HA^8^, adaptadas às necessidades dos países da América Central e do Sul, devem ser aplicadas e adotadas pela maioria dos médicos latino-americanos^21^. O conhecimento e a implementação dessas diretrizes representam um dos desafios mais significativos enfrentados pelas sociedades de HA na América Latina, como a Sociedade Centro-Americana e do Caribe de Hipertensão Arterial (SCCH), a Sociedade Latino-Americana de HA (LASH) e a Sociedade Interamericana de Cardiologia (SIAC). O documento da SIAC reforça todas as propostas das diretrizes da LASH no que se refere à abordagem terapêutica e às recomendações farmacológicas para pacientes com HA, a fim de alcançar um melhor controle da HA na região da América Central e do Caribe e, assim, melhorar o prognóstico de doenças cardiovasculares na região^6,8,13,22^.

Para atingir os objetivos primários do trata­mento anti-hipertensivo, ou seja, a redução da morbimortalidade cardiovascular e renal, bem como uma melhor qualidade de vida, é imprescindível que o tratamento seja iniciado prontamente (idealmente antes da ocorrência de danos significativos aos órgãos), seja eficaz (atingindo as metas prescritas) e seja mantido ao longo do tempo (com metas consistentemente atingidas). Alcançar essas metas é um desafio significativo em escala global, com a América Latina enfrentando obstáculos particularmente formidáveis.

Os desafios para os países da ALC, uma das regiões do mundo com as disparidades mais significativas em termos de condições socioeconômicas, estão relacionados aos contrastes no acesso à saúde. A proporção de pessoas que vivem em situação de pobreza varia consideravelmente entre as diferentes regiões dos países da ALC^6^. Além disso, existem disparidades significativas com relação à estrutura, acessibilidade, qualidade e financiamento dos sistemas nacionais de saúde. Alguns sistemas são totalmente públicos, como o sistema cubano, ou predominantemente públicos, como os da Jamaica^10^. Além disso, existem muitos sistemas mistos, como é o caso do Brasil, Equador e Peru. Na região, há disparidades significativas na equidade dos serviços médicos. Em países como Bolívia, Peru e Guatemala, apenas 19,8%, 14,3% e 9,3%, respectivamente, dos indivíduos de baixa renda têm acesso a serviços médicos^34^.

É de extrema importância realizar com precisão a medição da PA em consultório para o diagnóstico e o manejo eficaz da HA. Essa questão é mais urgente na América Latina do que em qualquer outra parte do mundo^38^. Na América Latina, a medição da PA no consultório (PAC) é predominantemente utilizada para triagem, diagnóstico, monitoramento e acompanhamento da HA. No entanto, tem havido um aumento da conscientização sobre as limitações da PAC, e as evidências do valor da monitorização ambulatorial da PA (MAPA) como complemento da PAC na abordagem clínica do paciente hipertenso têm se acumulado e representam o padrão-ouro para as medições da PA^38,39,43^. Os padrões de PA diurna e noturna oferecidos pela MAPA de 24 horas no manejo diagnóstico, prognóstico e terapêutico da HA têm ganhado destaque mundialmente. Nos países da América Latina, a maioria das sociedades científicas de HA e/ou cardiologia publicou diretrizes para o tratamento da HA, três das quais incluem uma seção dedicada à MAPA. Entretanto, na América Latina, o acesso à MAPA é frequentemente restrito a áreas urbanas com grande densidade populacional. Além disso, os custos associados a essa tecnologia podem impedir sua utilização por pacientes com recursos financeiros limitados, especialmente nos países da ALC^38,43–46^. Outra questão crítica nos países da ALC é que cada um deles possui sua própria agência nacional reguladora para a validação de dispositivos médicos. Uma política uniforme em todos os países da ALC para a utilização do mesmo protocolo internacional de validação deve contribuir para reduzir o uso de monitores de PA imprecisos no mercado^8^.

### Perspectivas

Em conformidade com o *World Heart Federation Roadmap for HT* ^47^, uma série de ações tem sido proposta, incluindo o desenvolvimento de programas de prevenção e controle em toda a população, a implementação de triagem oportunista e medições de PA fora do consultório. O fortalecimento da atenção primária e uma ênfase maior no compartilhamento de tarefas e no atendimento em equipe são essenciais. A prestação de cuidados centrados nas pessoas e a educação voltada ao paciente e aos cuidados, juntamente com a promoção da adesão ao tratamento, são fundamentais^47^. Todos os aspectos acima dependem da disponibilidade e da distribuição eficaz de agentes redutores da PA de boa qualidade, baseados em evidências e de baixo custo.

O uso da telemedicina, aliado às medições da PA fora do consultório médico, pode melhorar o acesso ao sistema de saúde, a adesão e a manutenção do tratamento da HA^48^. A introdução de sistemas de telemonitoramento da PA pode melhorar a triagem de rotina e o manejo da HA após seu diagnóstico no consultório e, consequentemente, reduzir o impacto adverso na carga global da doença na América Latina. Uma questão transcendental é a necessidade de conscientizar a população sobre os benefícios da aferição da PA como forma de evitar complicações da HA e, assim, promover a criação de mecanismos de teleconsulta para o acompanhamento de pacientes já diagnosticados com a doença.

Atualmente, os sistemas de inteligência artificial também têm sido utilizados no desenvolvimento de novas tecnologias de monitoramento da PA, que consistem em sensores vestíveis, com ou sem manguito, e utilizam sensores mecânicos e ópticos para determinar as características da forma de onda do pulso sanguíneo, como tonometria, fotopletismografia e capacitância, a fim de estimar a PA^8^.

Por fim, as lições aprendidas com experiências bem-sucedidas em países de alta renda podem ser adaptadas regionalmente e promover o intercâmbio de recursos, tecnologia e conhecimento entre países de baixa e média renda. O principal objetivo é atingir as metas da Agenda 2030 das Nações Unidas para o Desenvolvimento Sustentável^4^.

## Data Availability

Nenhum dado novo foi gerado ou analisado neste estudo.
